# Outcomes of Hybrid Cement-Augmented Pedicle Screw Fixation in Complicated Osteoporotic Thoracolumbar Fractures: A Single-Centre Experience

**DOI:** 10.3390/medicina62030573

**Published:** 2026-03-19

**Authors:** Nurzhan Abishev, Talgat Kerimbayev, Daryn Borangaliyev, Galymzhan Kadirbekov, Zhandos Tuigynov, Yermek Urunbayev, Meirzhan Oshayev, Viktor Aleinikov, Yergen Kenzhegulov, Medet Toleubayev, Mariya Dmitriyeva, Makar Solodovnikov, Serik Akshulakov

**Affiliations:** 1 Department of Spinal Neurosurgery and Peripheral Nervous System Pathology, National Center for Neurosurgery, Astana 010000, Kazakhstan; dr.nb.abishev@gmail.com (N.A.); prof.kerimbayev.neurosurg@gmail.com (T.K.); kadirbekovgalymzan@gmail.com (G.K.); dr.tuigynov@gmail.com (Z.T.); doctor.urunbayev@gmail.com (Y.U.); dr.neurosurgeon@mail.ru (M.O.); doctor.aleynikov@gmail.com (V.A.); dr.kenzhegulov@gmail.com (Y.K.); solodovnikov.makar84@gmail.com (M.S.); s.akshulakov@gmail.com (S.A.); 2 Department of Surgery, Astana Medical University, Astana 010000, Kazakhstan; toleubayev.m@amu.kz (M.T.); dmitriyeva.m@amu.kz (M.D.)

**Keywords:** osteoporotic vertebral compression fractures, hybrid stabilisation, cement augmentation, AO Spine-DGOU classification, thoracolumbar spine, surgical outcomes

## Abstract

*Background and Objectives:* Complicated osteoporotic thoracolumbar fractures represent a major surgical challenge because compromised bone quality predisposes to progressive deformity, neurological deterioration, and fixation failure. This study aimed to evaluate the clinical and radiological outcomes of hybrid stabilization in patients with severe osteoporotic fractures classified as AO Spine-DGOU OF4–OF5. *Materials and Methods:* This single-center retrospective observational cohort study included 87 consecutively treated patients with complicated osteoporotic thoracolumbar fractures who underwent surgical treatment between 2012 and 2022. Clinical outcomes were assessed using the Visual Analog Scale (VAS) and Oswestry Disability Index (ODI). Radiological outcomes included the regional kyphotic angle (RKA) and interbody fusion graded according to the Bridwell classification. Imaging was reviewed preoperatively, immediately postoperatively, and at follow-up, with 12-month outcomes used for the principal analysis. Additionally, a retrospective comparative analysis was undertaken between the two largest fixation subgroups within the cohort to explore outcome differences across the most representative construct patterns. *Results:* At 12 months, complete interbody fusion (Bridwell grade I) was achieved in 75.9% of patients. Mean RKA improved from 29.4° ± 14.1° preoperatively to 7.9° ± 8.0° immediately after surgery, with only minimal loss of correction during follow-up. Mean VAS improved from 7.0 ± 1.8 to 2.1 ± 1.2, while mean ODI decreased from 61.3% ± 6.8% to 9.8% ± 1.2% (both *p* < 0.001). Reoperation for implant-related mechanical failure was required in three patients (3.4%). *Conclusions:* Hybrid stabilization with cement augmentation was associated with marked improvement in pain, functional disability, and sagittal alignment, as well as a high rate of interbody fusion at 12 months, in patients with complicated osteoporotic thoracolumbar fractures. Given the retrospective observational design, these findings should be interpreted as associations within the treated cohort. Prospective comparative studies are warranted to further validate these results.

## 1. Introduction

As the global population ages, osteoporotic vertebral fractures (OVFs) have become an increasingly common cause of acute pain, functional decline, and progressive sagittal malalignment, occasionally complicated by neurological deficit and loss of independence [1,2,3]. The prevalence of vertebral fractures rises sharply with age, affecting a substantial proportion of older adults, particularly women, and the incidence continues to increase in parallel with population aging [4]. In many patients, even low-energy mechanisms can result in clinically significant fractures; when conservative management fails, ongoing collapse, kyphotic progression, and persistent pain may necessitate surgical intervention [1,5].

Surgical treatment in osteoporotic bone is technically demanding due to compromised screw purchase and a higher risk of fixation failure, progressive deformity, and junctional or adjacent-level complications [4,6,7,8]. These challenges highlight the need for a fracture classification that not only describes morphology but also provides clinically meaningful stratification of instability severity in osteoporotic bone. Several frameworks exist to describe vertebral fractures, including deformity-based grading systems and trauma-oriented classifications; however, many are not primarily tailored to osteoporotic failure patterns and may have limited utility for standardizing reporting of advanced OVFs with progressive collapse and posterior wall involvement [9].

To address this gap, the AO Spine–DGOU Osteoporotic Fracture (OF) classification was introduced as a morphology-based system developed specifically for OVFs, aiming to enable consistent categorization across the spectrum of osteoporotic fracture patterns (OF1–OF5) and to support structured clinical decision-making in severe or unstable presentations [3]. In particular, OF4–OF5 fractures represent advanced patterns with instability and/or posterior wall compromise that frequently prompt consideration of surgical stabilization.

In this context, we aimed to evaluate clinical and radiological outcomes of hybrid stabilization—cement-augmented transpedicular screw fixation with anterior/interbody support when indicated—in a single-center cohort of patients with thoracolumbar OVFs classified as AO Spine–DGOU OF4–OF5. We hypothesized that this strategy would be associated with meaningful pain and functional improvement and maintenance of radiological alignment, while acknowledging the inherent limitations of an observational retrospective design.

## 2. Materials and Methods

This study includes patients with complicated osteoporotic fractures localized to the thoracolumbar spine, treated at the Department of Spine Neurosurgery and Peripheral Nervous System Pathology of the JSC “National Center for Neurosurgery.”

This was a single-center retrospective observational cohort study at the National Center for Neurosurgery (Astana, Kazakhstan), including consecutively treated patients from 2012 to 2022. The cohort comprised 87 patients, both male and female, aged 45 to 82 years.

The study was approved by the local ethics committee of the National Center for Neurosurgery. Written informed consent for surgical treatment was obtained from all patients as part of routine clinical care. The requirement for individual informed consent for participation in this retrospective study was waived by the ethics committee due to the study design and analysis of anonymized data.

Fractures were classified using the AO Spine–DGOU Osteoporotic Fracture (OF) classification (OF1–OF5) based on morphologic severity and instability patterns [3]. This system was selected because it is intended specifically for osteoporotic vertebral fracture morphology and provides standardized stratification of advanced osteoporotic fracture patterns (including OF4–OF5), supporting consistent reporting and clinical decision-making in severe presentations.

### 2.1. Inclusion Criteria

Patients were included if they met all of the following: Thoracolumbar osteoporotic fracture classified as AO Spine–DGOU OF4 or OF5; Reduced bone quality on CT attenuation assessment (Hounsfield units, HU) consistent with osteopenia/osteoporosis; Complicated clinical presentation requiring surgery, including neurological symptoms (e.g., lower-limb paresis, paresthesia, or pelvic organ dysfunction) and/or progressive collapse/kyphotic deformity or persistent pain with functional decline despite conservative measures; Availability of baseline CT (±MRI) and postoperative imaging sufficient for radiological assessment.

Exclusion criteria: Uncorrectable coagulopathy; local or systemic infection (including spondylitis/spondylodiscitis); active systemic inflammation suggestive of infection (e.g., markedly elevated CRP and/or leukocytosis with clinical suspicion); spinal neoplasms unrelated to osteoporosis; pathological fractures due to tumor or infection; severe psychiatric disorders affecting compliance; decompensated cardiovascular or respiratory disease; or inability to adhere to postoperative follow-up.

### 2.2. Baseline Data and Perioperative Risk

For each patient, demographic and clinical characteristics were collected, including age, sex, body mass index (BMI), comorbidities (e.g., hypertension, diabetes mellitus, congestive heart failure, COPD, chronic infections), and perioperative anesthetic risk. Perioperative risk was graded using the American Society of Anesthesiologists (ASA) classification [10,11].

Neurological status was assessed preoperatively, at discharge, and at follow-up using standardized neurological examinations documented in the medical records. Functional neurological status was graded using the Frankel scale (A–E), and muscle strength was graded using the Medical Research Council (MRC) scale (0–5) for key lower-limb myotomes.

Radiological assessments were performed preoperatively, immediately postoperatively, and at 3 and 12 months. Parameters included fracture angle, vertebral body compression, and regional kyphotic angle. Fixation stability and interbody fusion were evaluated on CT scans according to the Bridwell grading system. Clinical outcomes were assessed using the Visual Analog Scale (VAS) for pain and the Oswestry Disability Index (ODI) for functional status. Operative time, intraoperative blood loss, and length of hospital stay were also recorded. The median follow-up was 12 months. Radiological measurements were performed by a spine surgeon according to a prespecified protocol; due to the retrospective nature of the study, formal interobserver reliability testing was not performed and is acknowledged as a limitation.

For CT-based bone quality assessment, the region of interest (ROI) was placed centrally within the trabecular bone while carefully excluding cortical margins and visible fracture lines to obtain cancellous attenuation in Hounsfield units (HU). Osteopenia was defined as 100–160 HU and osteoporosis as <100 HU based on CT attenuation values. The regional kyphotic angle was measured on sagittal CT reconstructions from the superior endplate of the vertebra above the fracture to the inferior endplate of the vertebra below.

**Figure 1:**
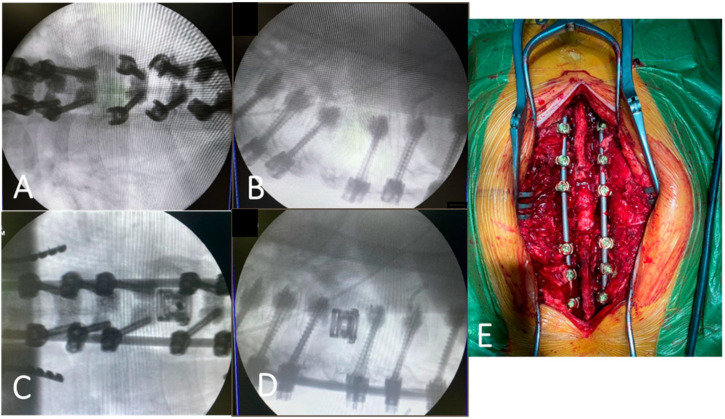
Intraoperative X-rays of implant placement (**A**–**D**) and intraoperative imaging (**E**). (**A**) Frontal radiograph after transpedicular screw fixation. (**B**) Sagittal radiograph after transpedicular screw fixation. (**C**) Frontal radiograph after cage replacement. (**D**) Sagittal radiograph after cage replacement. (**E**) Posterior intraoperative view.

All procedures were performed under general anesthesia with the patient in the prone position. Posterior exposure was obtained via a midline incision with subperiosteal muscle dissection. Pedicle screw instrumentation was performed under fluoroscopic guidance using cannulated fenestrated screws, followed by cement augmentation (Vertplex, Stryker, Kalamazoo, MI, USA) to improve fixation in osteoporotic bone. Decompression and vertebral body reconstruction were performed when indicated, including adjacent-level discectomy and anterior column support using either a titanium mesh cage or an expandable V-LIFT cage (Stryker, Kalamazoo, MI, USA). Implant position and alignment restoration were confirmed intraoperatively using fluoroscopy. Blood conservation strategies (including autotransfusion) were applied in patients at increased risk of blood loss. Hemostasis was achieved using standard techniques and topical hemostatic agents as required; layered wound closure and suction drainage were performed. Key operative steps are illustrated in Figure 1.

All patients received pharmacological therapy for osteoporosis according to national guidelines. Regular bone density monitoring and adherence to therapy formed the basis of the multidisciplinary management approach.

### 2.3. Outcomes

Outcome assessment was purposefully focused on parameters reflecting the mechanical performance and stability of the fixation construct. Given the retrospective study design and the considerable comorbidity burden of the study population, a comprehensive evaluation of all perioperative and general medical complications was beyond the methodological scope of the present analysis. Therefore, the assessment was limited to predefined variables directly related to the study objectives and consistently available for serial evaluation throughout follow-up. The main variables of interest were construct length and the use of cement augmentation. The primary outcome measures were radiographic indicators of construct stability and fusion, including the kyphotic angle and fusion grade according to the Bridwell classification. Clinical outcomes were additionally assessed using the Visual Analog Scale (VAS) for pain and the Oswestry Disability Index (ODI) for functional status.

No a priori sample size calculation was performed because this was a retrospective consecutive-case cohort; the sample size was determined by the number of eligible patients treated during the study period.

### 2.4. Statistical Analysis

Statistical analyses were performed in Python version 3.11 (Python Software Foundation, Wilmington, DE, USA). The full cohort (N = 87) was used for descriptive statistics. For comparative analyses, we focused on the two most frequent posterior fixation configurations (8 screws, n = 39; 12 screws, n = 25; total n = 64) to ensure adequate sample size; the remaining construct sizes (10, 14, and ≥16 screws; n = 23) were reported descriptively only and were not included in inferential comparisons due to small subgroup sizes.

Continuous variables are presented as mean ± standard deviation (SD), and categorical variables as counts and percentages. Normality of change scores was assessed using the Shapiro–Wilk test and Q–Q plots. Because several outcomes deviated from normality, between-group comparisons at each timepoint were performed using the Mann–Whitney U test, and within-group changes from baseline to 12 months were assessed using the Wilcoxon signed-rank test.

To account for baseline imbalances, 12-month outcomes (VAS, ODI, and regional kyphotic angle) were additionally analyzed using baseline-adjusted analysis of covariance (ANCOVA; linear regression with the baseline value as a covariate), reporting adjusted mean differences (12 vs. 8 screws) with 95% confidence intervals (CIs) and robust (HC3) standard errors.

Fusion status (Bridwell grade I vs. II–IV) and other categorical outcomes were compared using Fisher’s exact test; odds ratios (ORs) with 95% CIs are reported.

All tests were two-sided, and *p* < 0.05 was considered statistically significant.

## 3. Results

Eighty-seven patients with complicated osteoporotic thoracolumbar fractures treated between 2012 and 2022 were included (N = 87). The cohort was predominantly female (62/87, 71.3%); mean age was 63.1 ± 6.7 years (range, 45–82). Most patients were 60–69 years old (48/87, 55.2%). Baseline characteristics are summarized in Table 1.

### 3.1. Overall Outcomes

#### 3.1.1. Perioperative Outcomes

Mean operative time was 169.5 min (range, 150–240), mean intraoperative blood loss was 570 mL (range, 200–1550), and mean length of stay was 10.4 ± 1.6 days. Most patients were mobilized within 24 h; mobilization was delayed in selected cases due to comorbidities. Twelve-month outcomes are reported for the analytic cohort with available data (complete-case analysis).

#### 3.1.2. Pain Outcomes

**Figure 2:**
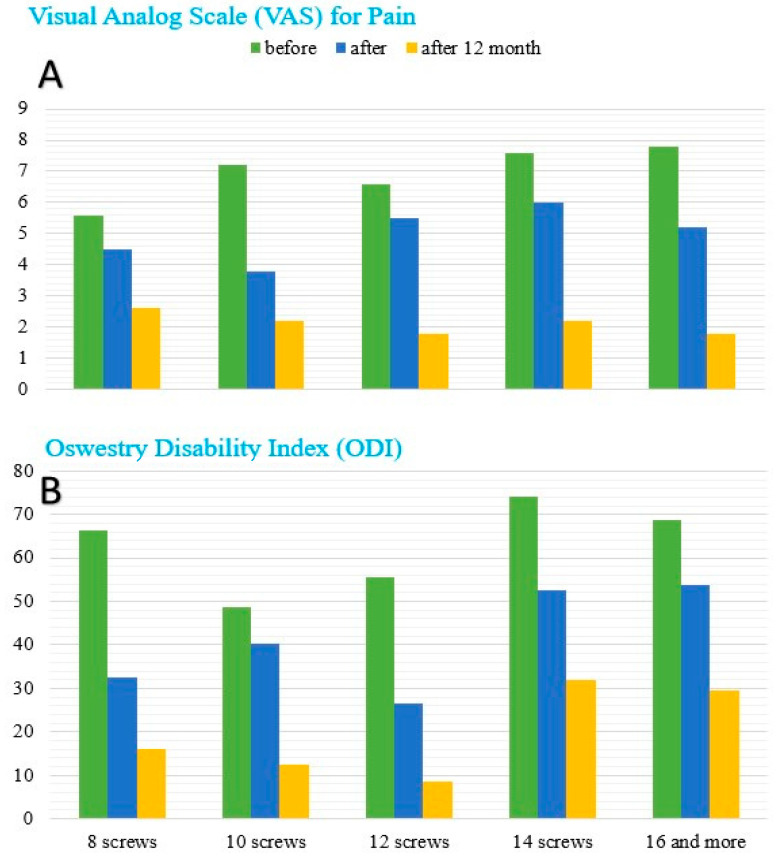
Changes in clinical outcomes after surgery. (**A**) Visual analog scale (VAS) scores for pain measured preoperatively, at discharge, and at 12 months. (**B**) Oswestry Disability Index (ODI) scores assessed at the same time points, stratified by the number of pedicle screws (8, 10, 12, 14, ≥16).

Mean VAS decreased from 7.0 ± 1.8 preoperatively to 5.0 ± 1.3 at discharge (*p* < 0.001) and to 2.1 ± 1.2 at 12 months (*p* < 0.001), indicating sustained analgesic benefit (Figure 2A). The 95% CIs for mean VAS were 6.62–7.38 (baseline), 4.72–5.28 (discharge), and 1.84–2.36 (12 months), respectively.

#### 3.1.3. Functional Outcomes

ODI improved from 61.3 ± 6.8% preoperatively to 27.8 ± 4.7% at discharge and 9.8 ± 1.2% at 12 months (both *p* < 0.001) (Figure 2B). The 95% CIs for mean ODI were 59.85–62.75 (baseline), 26.80–28.80 (discharge), and 9.54–10.06 (12 months). In the prespecified subgroup analysis by construct size, the lowest 12-month ODI was observed in the 12-screw subgroup (n = 25, 8.6), whereas comparatively higher values were noted in the 14-screw (n = 5, 32.0) and ≥16-screw (n = 5, 29.5) subgroups, consistent with greater baseline disease burden in these patients. Given small subgroup sizes, these comparisons are descriptive.

**Comparative analysis: 8 vs. 12-screw constructs.**

In the comparative cohort (n = 64), baseline VAS was higher in the 12-screw group than in the 8-screw group (6.6 ± 1.4 vs. 5.6 ± 1.4; *p* = 0.014), whereas baseline ODI was lower (55.6 ± 5.1 vs. 66.2 ± 8.7; *p* < 0.001) and baseline regional kyphotic angle (RKA) was higher (28.2° ± 2.6° vs. 23.2° ± 4.9°; *p* < 0.001). Both groups demonstrated significant improvement from baseline to 12 months for VAS, ODI, and RKA (all Wilcoxon *p* < 0.001).

At 12 months, the 12-screw group had lower VAS (1.8 ± 0.9 vs. 2.6 ± 1.2; *p* = 0.003), lower ODI (8.6 ± 3.3 vs. 16.2 ± 4.8; *p* < 0.001), and lower residual RKA (7.8° ± 1.0° vs. 9.0° ± 0.7°; *p* < 0.001) compared with the 8-screw group (Table 2). After baseline adjustment (ANCOVA), differences at 12 months remained significant for VAS (adjusted mean difference −0.76, 95% CI −1.32 to −0.21; *p* = 0.007), ODI (−6.65, 95% CI −8.93 to −4.38; *p* < 0.001), and RKA (−0.93°, 95% CI −1.42 to −0.45; *p* < 0.001) (Table 3).

Interbody fusion (Bridwell). At 12 months, fusion status varied by construct size (Table 4). Grade 1 fusion occurred in 23/25 patients in the 12-screw subgroup. Grade 2 was most frequent in the 8-screw subgroup (10/39) and was observed in 2/13 patients in the 10-screw subgroup and 2/25 patients in the 12-screw subgroup. Grades 3–4 were confined to the 8-screw subgroup (5/39 and 1/39, respectively) and to 1/13 patients in the 10-screw subgroup (Grade 4). The 14-screw and ≥16-screw subgroups each showed 100% Grade 1 fusion (5/5 and 5/5); however, these subgroups were small, and findings should be interpreted cautiously. In the comparative cohort, complete fusion (Grade 1) was more frequent in the 12-screw group (23/25, 92.0%) than in the 8-screw group (23/39, 59.0%; Fisher’s exact *p* = 0.004). The odds of incomplete fusion (Bridwell II–IV) were higher with 8-screw constructs (OR 8.0, 95% CI 1.65–38.82).

At 12 months, patients with osteopenia (n = 20) achieved Grade 1 fusion in 18/20 (90.0%), whereas those with osteoporosis (n = 67) demonstrated lower consolidation rates (Grade 1: 46; Grade 2: 13; Grade 3: 5; Grade 4: 3), suggesting an association between lower CT-based Hounsfield units and less favorable fusion status.

Assessment of the regional kyphotic angle was performed on sagittal CT reconstructions, measured from the superior endplate of the vertebra above the fracture to the inferior endplate of the vertebra below (Table 5). The mean preoperative angle was 29.4° ± 14.1°, which improved to 7.9° ± 8.0° postoperatively (*p* < 0.001). At 12 months, a minor loss of correction was observed in most groups. In the comparative cohort, the 12-screw group had a higher baseline RKA but a lower residual RKA at 12 months (7.8° ± 1.0° vs. 9.0° ± 0.7°; *p* < 0.001). After baseline adjustment, the 12-screw group remained associated with a lower 12-month RKA (adjusted difference −0.93°, 95% CI −1.42 to −0.45; *p* < 0.001).

**Figure 3:**
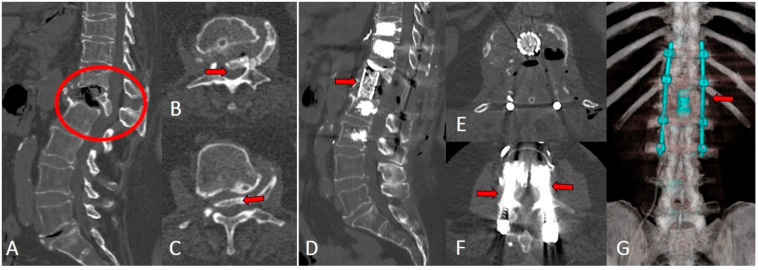
Representative imaging of a patient with an unstable OF5 fracture before (**A**–**C**) and after surgery (**D**–**G**). (**A**) Sagittal CT reconstruction showing a comminuted vertebral fracture with compression of the spinal canal (red circle). (**B**,**C**) Axial CT images demonstrating displaced bone fragments within the canal (arrows). (**D**) Sagittal CT reconstruction after placement of a titanium mesh cage (arrow) filled with autologous bone, showing restoration of vertebral height and formation of spondylodesis. (**E**) Axial CT view of the interbody cage positioned centrally. (**F**) Axial CT image showing bilateral transpedicular screw placement (arrows). (**G**) Three-dimensional CT reconstruction demonstrating correction of thoracolumbar alignment and coronal balance after cage insertion and posterior instrumentation.

**Illustrative cases**. A 59-year-old female patient presented with persistent low back pain that developed three months earlier after lifting a heavy object. The pain progressively worsened and was accompanied by lower extremity paresis. MRI in T2-weighted sequences showed signal reduction within the T8 vertebral body, indicating advanced structural compromise. CT confirmed the collapse of the T8 superior endplate with associated local kyphotic deformity. Postoperative CT demonstrated adequate posterior decompression, anterior spondylodesis with a V-LIFT cage, cement-augmented transpedicular screw fixation, restoration of vertebral height, correction of the kyphotic deformity, and decompression of the spinal canal (Figure 3).

**Figure 4:**
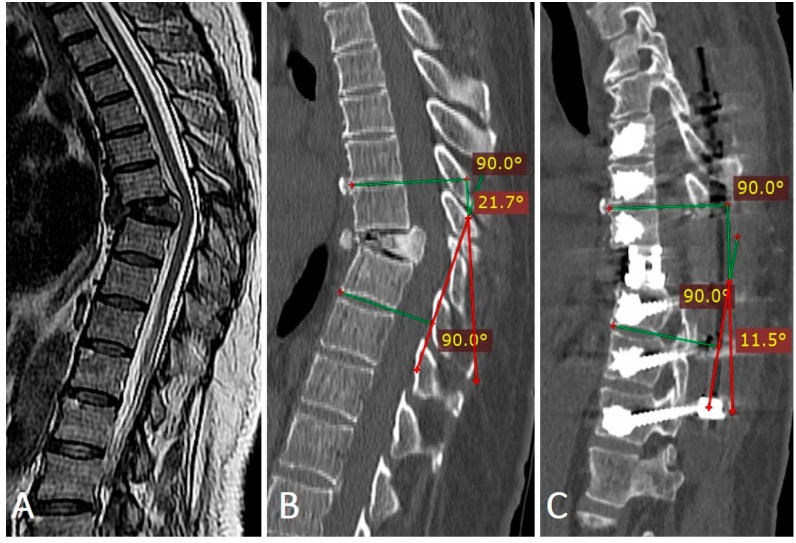
Preoperative (**A**,**B**) and postoperative (**C**) imaging of a 59-year-old female patient with an OF5 fracture of the T8 vertebra. (**B**) Preoperative sagittal view demonstrates a regional kyphotic angle of 21.7° (red angle). (**C**) Postoperative imaging shows effective deformity correction with reduction of the regional kyphotic angle to 11.7° (red angle).

In cases of unstable spinal fractures classified as OF4 or OF5 according to the AO Spine-DGOU system, transpedicular fixation of the injured and adjacent vertebrae was performed to enhance stability. Imaging findings in a representative patient with an OF5 fracture (Figure 4) demonstrated restoration of vertebral body height and complete resolution of spinal canal stenosis after a combined procedure consisting of posterior decompression, anterior spondylodesis, and cement-augmented transpedicular fixation.

### 3.2. Complications

**Figure 5:**
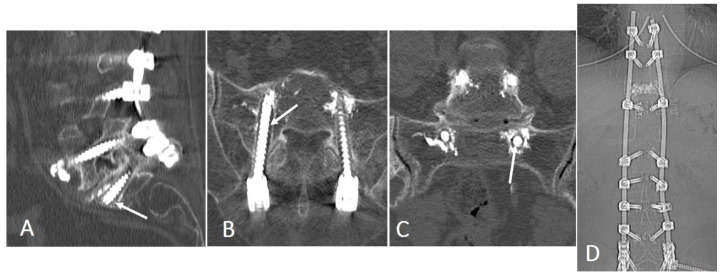
Postoperative CT images. (**A**–**C**) Axial and sagittal views showing bilateral S1 screw loosening with circumferential osteolysis of the surrounding bone (arrows). (**D**) Extended reconstruction demonstrating the implanted hardware in situ.

Three patients (3/87, 3.4%) required reoperation for implant-related failure associated with peri-screw osteolysis; extension of instrumentation restored stability in all cases (Figure 5). The 95% Wilson confidence interval for the reoperation rate was 1.18–9.65%. No other reoperation indications were identified during the 12-month follow-up in this cohort. Minor medical complications, asymptomatic cement leakage, and non-reoperative perioperative adverse events were not systematically captured in a standardized manner in this retrospective dataset and, therefore, were not analyzed as formal study outcomes.

## 4. Discussion

Osteoporotic compression fractures remain a major clinical challenge in elderly patients because of reduced bone mineral density and fracture instability. The present study demonstrates that the AO Spine-DGOU classification provides a practical framework for stratifying severe fractures (OF4–OF5), supporting systematic assessment of instability and guiding surgical decision-making with improved clinical and radiological outcomes. Importantly, this analysis focused on complicated OFs associated with neurological deficits, which typically require surgical intervention.

Previous reports have shown that osteoporotic vertebral fractures are associated with impaired quality of life and increased long-term mortality in postmenopausal women, underscoring the need for timely management in aging populations [8,12]. The thoracolumbar junction is particularly vulnerable to collapse, often resulting in progressive deformity and neurological impairment [11]. OF4 fractures, characterized by >50% vertebral body loss and severe kyphosis, usually require posterior fixation, whereas OF5 fractures, with >75% collapse and marked deformity, demand combined anterior–posterior reconstruction for optimal outcomes [3].

Several surgical strategies have been evaluated for osteoporotic fractures with nonunion. Kanayama et al. reported an 80% success rate using anterior spinal reconstruction with instrumentation in patients with neurological deficits [13]. Matsuyama et al. demonstrated the utility of posterior spondylodesis combined with calcium-phosphate vertebroplasty [14], while Akata et al. observed improvement after posterior fusion without decompression in thoracolumbar OFs [15]. More recently, Saita et al. introduced a posterior osteotomy technique involving spinal shortening to correct kyphosis and reduce implant failure, which proved effective in patients with paraparesis [16]. Collectively, these studies highlight the diversity of approaches available, but also reinforce the need for reliable classification systems to select the most appropriate surgical strategy.

Our exploratory comparison of the two most common construct configurations (8 vs. 12 screws) showed an association between the 12-screw construct and more favorable 12-month radiological and clinical outcomes; however, because construct length was not randomized, these findings should not be interpreted as evidence that a higher screw count itself caused better outcomes. Complete fusion (Bridwell Grade 1) at 12 months was more frequent in the 12-screw group (92.0%) than in the 8-screw group (59.0%; Fisher’s exact *p* = 0.004), with higher odds of incomplete fusion (Grades II–IV) observed with 8-screw constructs (OR 8.0, 95% CI 1.65–38.82). Notably, the 12-screw group demonstrated higher baseline pain and greater deformity, and junctional involvement was more common, indicating potential confounding by indication; therefore, these findings should be interpreted as associations rather than causal effects.

Bone quality, assessed using HU, also influenced fusion. Schreiber et al. reported a significant correlation between HU values and bone mineral density [17], while Florian et al. demonstrated the ability of HU to differentiate bone density independent of the cortical layer (R = 0.72) [18]. Consistent with these findings, patients in our series with osteopenia (100–160 HU) showed higher fusion rates (90% Grade 1) compared with those with osteoporosis (<100 HU), who had a greater incidence of incomplete fusion (Grade 1: 46, Grade 2: 13, Grade 3: 5, Grade 4: 3). These findings suggest that lower HU values were associated with less favorable fusion status in this cohort; however, this observation should be interpreted cautiously because no multivariable adjustment for potential confounders was performed.

Correction of kyphotic deformity and preservation of sagittal alignment are clinically important radiographic goals in osteoporotic thoracolumbar fractures, as greater thoracolumbar kyphosis and global sagittal malalignment have been associated with worse pain, disability, and quality-of-life outcomes [19,20]. In the present study, both groups achieved substantial immediate correction, whereas the 12-screw group demonstrated lower residual kyphosis at 12 months. This pattern is broadly consistent with prior comparative studies suggesting that more robust or longer-segment posterior fixation may provide better maintenance of kyphotic correction and lower mechanical complication rates, although the available literature is not directly equivalent to an 8- versus 12-screw construct comparison [21,22,23]. After baseline adjustment, the 12-screw configuration remained associated with a modestly lower 12-month RKA (adjusted difference −0.93°, *p* < 0.001). Clinically, this was paralleled by lower 12-month VAS and ODI values, although these associations should be interpreted cautiously given the retrospective design and the possibility of residual confounding.

Although cement-augmented transpedicular fixation with interbody cages remains an effective treatment for osteoporotic fractures, patient-specific 3D-printed implants may represent a future alternative. Biomechanical and early clinical studies have demonstrated their potential to improve stability and fusion after en bloc spondylectomy [24]. In cases of severe vertebral destruction or poor bone quality, such implants may optimize load distribution, enhance osseointegration, and reduce hardware failure, offering a promising strategy for improving outcomes in this high-risk population.

This study has several limitations. First, it was a retrospective single-center cohort study, which may introduce selection bias and limit generalizability. Second, the absence of a parallel control group precludes comparative assessment of hybrid cement-augmented fixation versus other surgical strategies; therefore, the findings should be interpreted as observational associations within the treated cohort rather than evidence of superiority. Third, construct length and screw number were not randomized and may have been influenced by fracture severity, junctional involvement, bone quality, and surgeon preference, introducing potential confounding by indication. In addition, several fixation subgroups were small, and baseline differences between groups may have affected the subgroup comparisons. Finally, although lower HU values were associated with less favorable fusion-related outcomes, no multivariable adjustment was performed; thus, this finding should be interpreted cautiously.

## 5. Conclusions

In this single-center retrospective cohort of complicated osteoporotic thoracolumbar fractures (OF4–OF5), hybrid stabilization with cement augmentation was associated with favorable clinical and radiological outcomes during follow-up. In the exploratory subgroup analysis, 12-screw constructs were associated with higher complete fusion rates and lower 12-month VAS, ODI, and residual kyphosis than 8-screw constructs after baseline adjustment; however, these differences should not be interpreted as causal because of the non-randomized design and potential confounding. Further prospective studies with standardized fixation strategies and longer follow-up are required.

## Figures and Tables

**Table 1:** Demographic and clinical characteristics of the study population.

Variables		Value, N (%)
**Age (years)**	45–50	3 (3.4)
	50–59	21 (24.1)
	60–69	48 (55.2)
	≥70	15 (17.2)
**Sex**	Men	25 (28.7)
	Women	62 (71.3)
**Body mass index (kg/m^2^)**	18.5–24.9	30 (34.5)
	25.0–29.9	34 (39.1)
	30.0–34.9	11 (12.6)
	35.0–39.9	7 (8.0)
	≥40	5 (5.7)
**Bone mineral density (HU)**	Osteopenia (100–160 HU)	20 (23.0)
	Osteoporosis (<100 HU)	67 (77.0)
**Fracture level**	T8	7 (8.0)
	T9	7 (8.0)
	T11	19 (21.8)
	T12	23 (26.4)
	L1	7 (8.0)
	L2	8 (9.2)
	L4	5 (5.7)
	multi-level	11 (12.6)
**DGOU fracture type**	OF 4	23 (26.4)
	OF 5	64 (73.6)
**Comorbidities**	Arterial hypertension	79 (90.8)
	Diabetes mellitus	36 (41.4)
	Chronic heart failure	33 (37.9)
	COPD	7 (8.0)
	rheumatoid disease	5 (5.7)

**Table 2:** VAS, ODI, and regional kyphotic angle (RKA) at baseline, immediate postoperative, and 12 months in the 8- and 12-screw groups.

Outcome	Timepoint	8 Screws (n = 39)	12 Screws (n = 25)	*p*
VAS	preop	5.6 ± 1.4	6.6 ± 1.4	0.014
VAS	postop	4.5 ± 1.2	5.5 ± 1.4	0.006
VAS	12 month	2.6 ± 1.2	1.8 ± 0.9	0.003
ODI (%)	preop	66.2 ± 8.7	55.6 ± 5.1	<0.001
ODI (%)	postop	32.5 ± 14.9	26.5 ± 2.8	0.093
ODI (%)	12 month	16.2 ± 4.8	8.6 ± 3.3	<0.001
RKA (°)	preop	23.2 ± 4.9	28.2 ± 2.6	<0.001
RKA (°)	postop	5.8 ± 1.3	6.8 ± 1.1	0.003
RKA (°)	12 month	9.0 ± 0.7	7.8 ± 1.0	<0.001

**Table 3:** Baseline-adjusted comparison of 12-month outcomes (ANCOVA) between the 12- and 8-screw groups.

Outcome (12 Months)	Adjusted Difference (12–8)	95% CI	*p*
**VAS**	−0.76	−1.32 to −0.21	0.007
**ODI**	−6.65	−8.93 to −4.38	<0.001
**RKA**	−0.93	−1.42 to −0.45	<0.001

**Table 4:** Assessment of spondylodesis at 12 months according to the Bridwell fusion grading system.

n	Screw Configuration	Grade 1	Grade 2	Grade 3	Grade 4
39	8 screws	23	10	5	1
13	10 screws	10	2	0	1
25	12 screws	23	2	0	0
5	14 screws	5	0	0	0
5	16 and more	5	0	0	0

**Table 5:** Dynamics of regional kyphotic angle correction according to the number of pedicle screws.

Screw Configuration	Before (°)	After (°)	At 12 Months (°)
8 screws	23.2	5.8	9
10 screws	24.4	7.4	8.7
12 screws	28.2	6.8	7.8
14 screws	26.6	10.6	13.5
≥16 screws	30.2	9.8	12

## Data Availability

The data presented in this study are available on request from the corresponding author. The data are not publicly available due to patient privacy and ethical restrictions.
